# Dissection of Closely Linked Quantitative Trait Locis Controlling Grain Size in Rice

**DOI:** 10.3389/fpls.2021.804444

**Published:** 2022-01-20

**Authors:** Pao Xue, Yu-yu Chen, Xiao-xia Wen, Bei-fang Wang, Qin-qin Yang, Ke Gong, Yi-wei Kang, Lian-ping Sun, Ping Yu, Li-yong Cao, Ying-xin Zhang, Xiao-deng Zhan, Shi-hua Cheng

**Affiliations:** ^1^Zhejiang Key Laboratory of Super Rice Research, State Key Laboratory of Rice Biology, China National Rice Research Institute, Hangzhou, China; ^2^Department of Resource and Environment, Moutai Institute, Renhuai, China

**Keywords:** rice, quantitative trait locus, grain size, small-effect, residual heterozygous population

## Abstract

Grain size is a key constituent of grain weight and appearance in rice. However, insufficient attention has been paid to the small-effect quantitative trait loci (QTLs) on the grain size. In the present study, residual heterozygous populations were developed for mapping two genetically linked small-effect QTLs for grain size. After the genotyping and the phenotyping of five successive generations, *qGS7.1* was dissected into three QTLs and two were selected for further analysis. The *qTGW7.2a* was finally mapped into a 21.10 kb interval containing four annotated candidate genes. Transcript levels assay showed that the expression of the candidates *LOC_Os07g39490* and the *LOC_Os07g39500* were significantly reduced in the NIL-*qTGW7.2a^BG1^*. The cytological observation indicated that *qTGW7.2a* regulated the grain width through controlling the cell expansion. Using the same strategy, *qTGW7.2b* was fine-mapped into a 52.71 kb interval containing eight annotated candidate genes, showing a significant effect on the grain length and width with opposite allelic directions, but little on the grain weight. Our study provides new genetic resources for yield improvement and for fine-tuning of grain size in rice.

## Introduction

Rice (*Oryza sativa* L.) is one of the most important staple crops which feeds half of the population of the world. Therefore, grain yield became a prime target for breeders. Grain yield is characterized by three components: panicle number, filled grain number per panicle, and grain weight. Grain weight is mainly determined by grain size, which simultaneously affects appearance (Zuo and Li, 2014). Thus, grain size is a primary target for yield improvement.

Grain length (GL) and grain width (GW) determine grain size, and both are complex traits controlled by the quantitative trait locus (QTL). To date, 20 grain size-related QTLs with large effects have been cloned and characterized. Several signals and regulatory pathways, controlling grain size and thousand-grain weight (TGW), have been identified in rice, such as the G-protein signaling pathway, the ubiquitin-proteasome pathway, the mitogen-activated protein kinase (MAPK) signaling pathway, the phytohormone signaling, and transcriptional regulators (Li and Li, 2016; Fan and Li, 2019). The *GS3* and *DEP1* encode G-protein γ-subunits and regulate the grain size and weight (Fan et al., 2006; Huang et al., 2009). The *OsLG3b* encodes a MADS-domain transcription factor OsMADS1, which acts as a key downstream effector of G-protein βγ dimers in controlling grain size and appearance (Yu et al., 2018). The *HGW*, *GW2*, *WTG1*, and *OsUBP15* regulate the grain size and weight via the ubiquitin-proteasome pathway (Song et al., 2007; Li et al., 2012; Huang et al., 2017; Shi et al., 2019). The *OsMKKK10*, *OsMKK4*/*SMG1*, and *OsMAPK6* are involved in the MAPK signaling pathway (Duan et al., 2014; Liu S. Y. et al., 2015; Guo et al., 2018). The OsRac1 and GSN1 directly interact with OsMAPK6, and also inactivate and activate the OsMAPK6 *via* dephosphorylation and phosphorylation (Guo et al., 2018; Zhang et al., 2019). Furthermore, *ERECTA1* acts upstream of the OsMKKK10-OsMKK4-OsMPK6 cascade to control the spikelet number by regulating cytokinin metabolism in rice (Guo et al., 2020). Some proteins participate in the brassinosteroids (BR) signal pathway. Particularly, *GW5* encodes a calmodulin-binding protein, *GS5* encodes a putative serine carboxypeptidase, *GL3.1* encodes a protein phosphatase kelch (PPKL), *GS2* encodes transcription factor OsGRF4, and GSK2 kinase has multiple substrates that carry out various BR responses (Li et al., 2011; Qi et al., 2012; Hu et al., 2015; Liu et al., 2017). In addition, *TGW6*, *BG1*, *GL3.3*/*TGW3*/*qTGW3*, *GSA1*, and *RBG1* are involved in the auxins signaling pathway (Ishimaru et al., 2013; Liu L. C. et al., 2015; Hu et al., 2018; Xia et al., 2018; Ying et al., 2018; Dong et al., 2020; Lo et al., 2020). The *GNP1* encodes the GA20ox1, and at the same time, increases the grain number and yield by increasing cytokinin activity. The *GW6* encodes a GA-regulated *GAST* family protein and positively regulates the grain width and weight through the gibberellins pathway (Wu et al., 2016; Shi et al., 2020). Additionally, many other major QTLs regulate the grain size and weight through the transcriptional levels, such as *GW8*, *GL7*/*GW7*, *GW6a*, *GLW7*, *GL4*, *OsLG3*, *GS9*, *GL6*, *TGW2*, and *SG3* (Wang et al., 2012; Song et al., 2015; Wang S. K. et al., 2015; Wang Y. X. et al., 2015; Si et al., 2016; Wu et al., 2017; Yu et al., 2017; Xiong et al., 2018; Zhao et al., 2018; Wang A. H. et al., 2019; Li et al., 2020; Ruan et al., 2020).

Small-effect QTLs also play important roles in regulating the grain size and are widely utilized in commercial rice varieties (Kinoshita et al., 2017). Many QTLs with small-effects are responsible for quantitative genetic variation. These QTLs are often unexpected based on prior knowledge of the trait or correspond to computationally predicted genes (Mackay et al., 2009). Therefore, it is beneficial to validate these small-effect QTLs for breeding. In recent years, more than 400 small-effect QTLs for grain size and weight were reported (Huang et al., 2013). However, only a few were fine-mapped or cloned. The *DTH2* encodes a CONSTANS-like protein that promotes heading by inducing the florigen genes *Hd3a* and *RFLT1* (Wu et al., 2013). Notably, *qTGW1.2b* regulates grain weight which encodes a VQ-motif protein OsVQ4 (Chan et al., 2020). A naturally varying QTL, the *qTGW12a*, which encodes the multidrug and the toxic compound extrusion (MATE) transporter, regulates grain weight in rice (Du et al., 2021). Validation and dissection of more small-effect QTLs could provide a large number of the candidate genes and would be beneficial for establishing the network-controlling grain weight and grain size in rice.

The residual heterozygous method (Du et al., 2008) was mainly used for QTL mapping in this study. Residual heterozygote populations, the genotypic compositions are showing heterozygosity of the target region and high homozygosity in the background. The progeny population obtained by selfing is equal to the natural near-isogenic line (NIL)-F_2_ population, which applies to the QTL validating, resolving, and fine-mapping. To date, a series of small-effect QTLs have been fine mapped using this method (Dong et al., 2018; Wang W. H. et al., 2019; Zhu et al., 2019; Zhang et al., 2020).

In a previous study, a set of backcross recombinant inbred lines, between the *indica* rice variety BG1 (Big Grain 1) and the *japonica* rice variety XLJ (Xiaolijing), were used for QTL mapping. There were significant differences in a wide range of traits, including the heading date (HD), plant height (PH), flag leaf length (FLL), flag leaf width (FLW), GL, GW, and TGW, between the two parents. A small-effect grain size QTL, the *qGS7.1*, which had considerable effect on GL, GW, and TGW has been identified on chromosome 7 (Xue et al., 2019). In the present study, the *qGS7.1* was dissected into three QTLs, named *qTGW7.1*, *qTGW7.2a*, and *qTGW7.2b*. Finally, the *qTGW7.2a* was located into a 21.10 kb region which controls the grain width and weight, while the *qTGW7.2b* was mapped to a 52.71 kb interval, which inversely affects the grain length and width, not grain weight.

## Materials and Methods

### Plant Materials

Five runs and a total of 23 residual heterozygous populations were used to map the target QTL in this study. The populations were derived from two BC_4_F_6_ individuals, which showed heterozygosity of the target region and high homozygosity in the background from the cross of XLJ/////XLJ////XLJ///XLJ//XLJ/BG1 (Supplementary Figure 1).

In the first run, two single plants with heterozygous regions of the *qGS7.1* were selected. These plants were then developed into two BC_4_F_7_ populations consisting of 137 plants (R7) and 142 plants (R8) used for QTL validation and mapping. New polymorphic markers were designed and used to test genotypes of these populations.

In the second run, six resultant BC_4_F_8_ populations, R9 to R14, consisting of 189, 193, 198, 151, 116, and 213 plants, respectively, were developed from six residual heterozygous BC_4_F_7_ single plants with updated target regions. Then, the BC_4_F_9_ population containing 3,989 individuals derived from the R9 population was constructed and was used for selecting the recombinants.

In the third run for the QTL validation and mapping, eleven single plants were selected from the BC_4_F_9_ generation to develop eleven BC_4_F_10_ populations, to be named R15 to R25, for a total of 794 plants.

In the fourth run, three homozygous NIL populations in the segregating region, namely, N1 to N3, were developed to validate the QTL. Two single plants without the *qTGW7.2b* target region were selected and selfed to develop two populations, named R26 and R27, which were made up of 209 and 223 plants, respectively. Meanwhile, a BC_4_F_11_ population, including 6,128 individuals derived from the R23 population, was constructed and used for further mapping.

In the fifth run, two single plants were selected from BC_4_F_11_ plants in the XP7-12-XP7-23 interval to develop progeny populations consisting of 233 (R28) and 98 (R29) plants for validation and fine-mapping of the *qTGW7.2a*. Later, two non-recombinants were selected from the R28 population and selfed to develop two NILs of the NIL-*qTGW7.2a^BG1^* and the NIL-*qTGW7.2a^XLJ^* for traits measurement.

### Field Experiments and Traits Measurement

Plants were grown at the field stations of the China National Rice Research Institute in Lingshui, Hainan province, and Fuyang, Zhejiang province. After harvesting, 300 dry seeds were randomly selected for measuring TGW (g), GL (mm), GW (mm), and the ratio of the grain length to width (RLW) using an automatic seed counting and analyzing instrument (Model SC-G, Wanshen Ltd., Hangzhou, China).

### DNA Extraction and Molecular Markers Development

Total DNA was extracted from fresh leaf samples by the cetyltrimethylammonium bromide (CTAB) method (Murray and Thompson, 1980). The PCR products were visualized on 8% non-denaturing polyacrylamide gels by silver staining. A total of 31 polymorphic DNA markers were used (Supplementary Table 1).

### RNA Extraction and Quantitative Reverse-Transcriptase Polymerase Chain Reaction

Total RNA was extracted from rice panicles using RNAprep pure Plant Kit (TIANGEN, Beijing, China). Quantitative reverse-transcriptase polymerase chain reaction (qRT-PCR) was performed using SYBR Premix Ex Taq II (TAKARA, Dalian, China). Data analysis used the 2^–ΔΔCt^ method, while the *UBQ10* was used as the internal reference to normalize the gene expression (Livak and Schmittgen, 2001). The qRT-PCR primers used in this study are listed in Supplementary Table 1.

### Cytological Observation

During the heading stage, young spikelet hulls of NIL-*qTGW7.2a^BG1^* and NIL-*qTGW7.2a^XLJ^* were fixed in 2.5% glutaraldehyde for 12 h at 4°C, and then dehydrated in serial graded ethanol (30, 50, 70, 80, 90, 95, and 100%) before finally preserved in 100% ethanol. The samples were dried in a Hitachi HCP-2 critical point drier, and the cell length and width of the inner glumes were observed by scanning electron microscopy (Hitachi SU-8010, Hitachi, Japan). ImageJ software was used to measure cell numbers and cell size.

### Data Analysis

Three genotypes were obtained after genotyping this population. Two homozygous genotype plants which carried alleles from XLJ and BG1 were used to detect the phenotypic differences by Student’s *t*-test. We deduce there was a QTL when *p* < 0.05. Subsequently, the heterozygous individual harboring the target QTL was used for developing a new residual heterozygous population.

All the analysis data, including the additive effect (*A*) and the proportion of phenotypic variance explained by the QTL (*R*^2^), were obtained from the Windows QTL Cartographer Version 2.5 software to estimate the genetic effects.

## Results

### Validation and Mapping of *qGS7.1*

**FIGURE 1:**
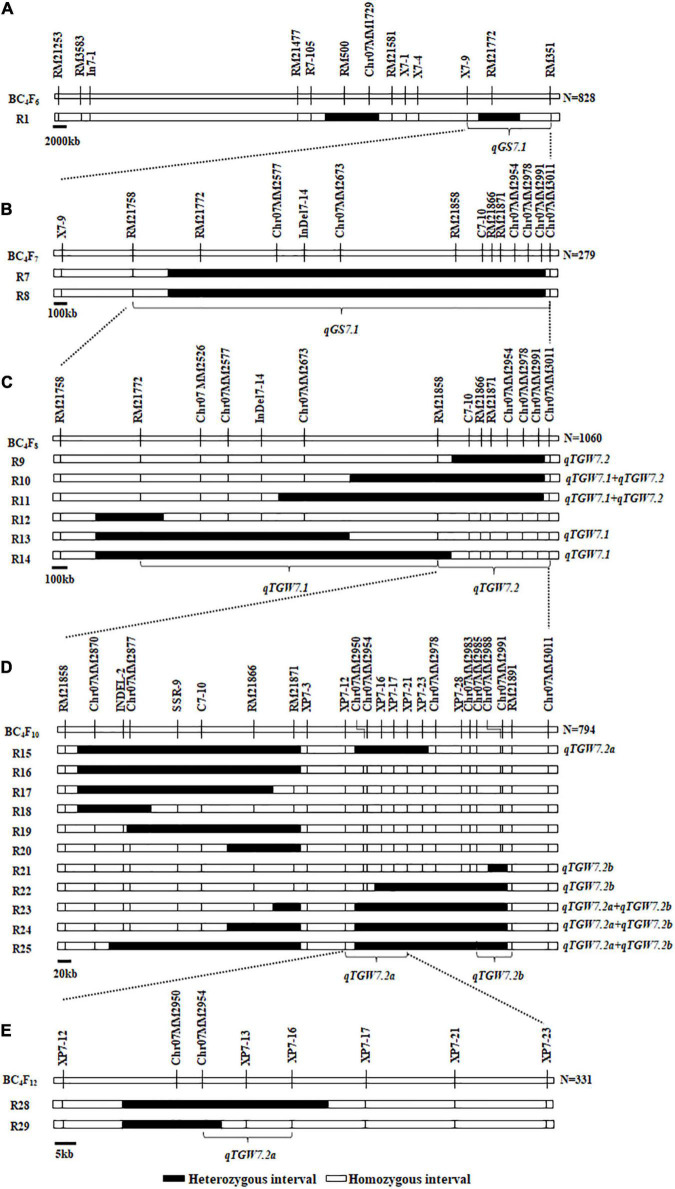
Genotypic compositions of residual heterozygous populations in the target regions. **(A)** Composition of R1 population in previous study. **(B)** Two residual heterozygous populations in BC_4_F_7_. **(C)** Six residual heterozygous populations in BC_4_F_8_. **(D)** Eleven residual heterozygous populations in BC_4_F_10_. **(E)** Two residual heterozygous populations in BC_4_F_12_.

We have identified a grain size QTL, the *qGS7.1*, in the X7-9-RM351 interval on chromosome 7 (Figure 1A). For further validation and fine-mapping of the target region, 12 polymorphic markers were designed based on the sequence differences between BG1 and XLJ. Consequently, the RM21758 became the new boundary when all plants were homozygous for it. Meanwhile, the R7 and R8 populations were derived from two segregated single plants selected from the R1 population (Figure 1B) to validate the *qGS7.1*, and to exclude the non-target interval in BC_4_F_7_ populations. Both showed a significant enhancement of GW, GL, and TGW between the two parental genotypic groups. These results showed that the *qGS7.1* with the XLJ allele have increased TGW, GL, and GW by 0.439–0.445 g, 0.075–0.082 mm, and.010–0.011 mm, and had *R*^2^ values of 21.95–28.01, 25.40–30.35, and 8.31–8.91%, respectively (Table 1). The effects detected in the two populations were comparable and indicated that the *qGS7.1* was located in the region between RM21758 and Chr07MM3011.

**TABLE 1:** Validation of *qGS7.1* using two residual heterozygous populations in BC_4_F_7_.

Name	Marker interval	Trait*^a^*	Phenotype (Mean ± SD)*^b^*		*P*	*A^c^*	*R*^2^ (%)*^d^*
			NIL*^XLJ^*	NIL*^BG1^*			
R7	RM21758-Chr07MM3011	TGW (g)	28.30 ± 0.579	27.43 ± 0.708	<0.0001	−0.445	21.95
		GL (mm)	8.897 ± 0.090	8.873 ± 0.091	<0.0001	−0.082	30.35
		GW (mm)	2.704 ± 0.027	2.681 ± 0.029	0.0024	−0.011	8.31
		RLW	3.303 ± 0.038	3.271 ± 0.035	0.0009	−0.015	9.53
R8	RM21758-Chr07MM3011	TGW (g)	26.77 ± 0.675	25.86 ± 0.506	<0.0001	−0.439	28.01
		GL (mm)	8.619 ± 0.088	8.478 ± 0.098	<0.0001	−0.075	25.40
		GW (mm)	2.669 ± 0.028	2.650 ± 0.032	0.0143	−0.010	8.91
		RLW	3.240 ± 0.034	3.212 ± 0.042	0.0052	−0.014	7.06

*^a^TGW, thousand grain weight (g); GL, grain length (mm); GW, grain width (mm); RLW, the ratio of grain length to width.*

*^b^NIL^XLJ^ and NIL^BG1^ are homozygous genotypes from XLJ and BG1 in the segregating region, respectively.*

*^c^A, additive effect, measured as the genetic effect when an XLJ allele is replaced by a BG1 allele.*

*^d^R^2^, proportion of phenotypic variance explained by the QTL.*

### Dissection of *qGS7.1* Into Three Quantitative Trait Loci

Six progeny populations with sequential segregating regions jointly covering the *qGS7.1* region (Figure 1C) were developed in BC_4_F_8_ populations. There were no significant differences in the R12 population (Table 2). In the remaining five populations, significant enhancements were discovered in XLJ alleles for TGW. The additive effects were −0.477, −0.670, −0.531, −0.265, and −0.180 g in R9, R10, R11, R13, and R14, respectively (Table 2). These results indicated that there were two hypotheses for the consistent allelic direction. Despite this, there were also significantly different effects among the five populations. Firstly, there were two QTLs for the TGW located in these populations. One was mapped in R9 but not in R13 and R14, while the other was segregated in R13 and R14 but not in R9. Both were located in R10 and R11, thus, the additive effects were higher in the two populations than those in the other populations. Secondly, only one QTL was segregated in these populations with highly unstable effects.

**TABLE 2:** Dissection of *qGS7.1* into two quantitative trait loci (QTLs) using six residual heterozygous populations in BC_4_F_8_.

Name	Marker interval	Trait*^a^*	Phenotype (mean ± SD)*^b^*		*P*	*A^c^*	*R*^2^ (%)*^d^*
			NIL*^XLJ^*	NIL*^BG1^*			
R9	RM21858-Chr07MM3011	TGW (g)	24.84 ± 0.503	23.85 ± 0.440	<0.0001	−0.477	32.37
		GL (mm)	8.908 ± 0.070	8.803 ± 0.063	<0.0001	−0.051	18.92
		GW (mm)	2.443 ± 0.035	2.417 ± 0.036	0.0026	−0.014	7.63
		RLW	3.659 ± 0.037	3.658 ± 0.051	0.9655		
R10	Chr07MM2673-Chr07MM3011	TGW (g)	25.42 ± 0.760	24.13 ± 0.807	<0.0001	−0.670	26.54
		GL (mm)	8.996 ± 0.185	8.768 ± 0.188	<0.0001	−0.114	14.27
		GW (mm)	2.482 ± 0.062	2.425 ± 0.062	<0.0001	−0.029	12.63
		RLW	3.638 ± 0.073	3.632 ± 0.084	0.7261		
R11	InDel7-14-Chr07MM3011	TGW (g)	24.96 ± 0.676	23.89 ± 0.828	<0.0001	−0.531	21.55
		GL (mm)	8.764 ± 0.100	8.640 ± 0.133	<0.0001	−0.063	13.96
		GW (mm)	2.484 ± 0.036	2.443 ± 0.036	<0.0001	−0.021	11.95
		RLW	3.542 ± 0.053	3.552 ± 0.050	0.3909		
R12	RM21758-Chr07mm2526	TGW (g)	23.68 ± 0.961	23.49 ± 0.715	0.3308		
		GL (mm)	8.815 ± 0.203	8.781 ± 0.182	0.4501		
		GW (mm)	2.493 ± 0.045	2.479 ± 0.039	0.1660		
		RLW	3.549 ± 0.067	3.555 ± 0.060	0.7012		
R13	RM21758-RM21858	TGW (g)	25.53 ± 0.641	25.01 ± 0.539	0.0006	−0.265	11.19
		GL (mm)	8.648 ± 0.187	8.633 ± 0.158	0.7100		
		GW (mm)	2.471 ± 0.072	2.410 ± 0.063	0.0005	−0.031	11.28
		RLW	3.513 ± 0.064	3.596 ± 0.068	<0.0001	0.042	20.21
R14	RM21758-C7-10	TGW (g)	25.43 ± 0.573	25.06 ± 0.800	0.0080	−0.180	4.06
		GL (mm)	8.829 ± 0.132	8.791 ± 0.179	0.2181		
		GW (mm)	2.519 ± 0.057	2.479 ± 0.082	0.0052	−0.018	7.25
		RLW	3.519 ± 0.069	3.561 ± 0.057	0.0034	0.021	5.78

*^a^TGW, thousand grain weight (g); GL, grain length (mm); GW, grain width (mm); RLW, the ratio of grain length to width.*

*^b^NIL^XLJ^ and NIL^BG1^ are homozygous genotypes from XLJ and BG1 in the segregating region, respectively.*

*^c^A, additive effect, measured as the genetic effect when an XLJ allele is replaced by a BG1 allele.*

*^d^R^2^, proportion of phenotypic variance explained by the QTL.*

For GL, significant effects were detected in three populations (Table 2) with the enhancing alleles always derived from XLJ. However, there were no significant differences between the two parental genotypic groups in R13 and R14 populations. To sum up, it could be concluded that the *qGS7.1* was a composite of two independent QTLs (Figure 1C). The first QTL, named *qTGW7.1*, was located in the common segregating region of R13 and R14, with the allele from XLJ increasing the TGW and GW. The second QTL, named *qTGW7.2*, was located in the heterozygous interval in R9, with the allele from XLJ increasing the TGW, GL, and GW.

Finally, the *qTGW7.2* was selected for further analysis. Eleven populations (R15-R25) were developed from 11 heterozygous individuals in BC_4_F_9_ populations (Figure 1D). Significant differences were found in the TGW, GL, and GW with the positive allele from XLJ in the R23, R24, and R25 populations. The genotypic effects were similar to the *qTGW7.2* (Table 3). In the R15 population, significant genotypic effects were detected in TGW and GW, but not in GL. In R21 and R22 populations, similar additive effects for GL were −0.050 and −0.053 mm, respectively explaining the 8.32 and 20.81% of the genotypic variance in both populations. There was no significant difference in R16, R17, R18, R19, and R20 between the two parental genotypic groups (Table 3).

**TABLE 3:** Dissection of *qTGW7.2* into two QTLs using eleven residual heterozygous populations in BC_4_F_10_.

Name	Marker interval	Trait*^a^*	Phenotype (mean ± SD)*^b^*		*P*	*A^c^*	*R*^2^ (%)*^d^*
			NIL*^XLJ^*	NIL*^BG1^*			
R15	Chr07MM2673-Chr07MM2978	TGW (g)	25.82 ± 0.596	24.61 ± 0.387	<0.0001	−0.604	42.48
		GL (mm)	9.100 ± 0.049	9.060 ± 0.057	0.1038		
		GW (mm)	2.534 ± 0.028	2.466 ± 0.022	<0.0001	−0.034	49.12
		RLW	3.609 ± 0.040	3.692 ± 0.029	<0.0001	0.041	42.01
R16	Chr07MM2673-Chr07MM2954	TGW (g)	25.32 ± 0.496	25.40 ± 0.349	0.523		
		GL (mm)	9.215 ± 0.061	9.254 ± 0.071	0.0753		
		GW (mm)	2.492 ± 0.030	2.494 ± 0.031	0.8173		
		RLW	3.716 ± 0.051	3.728 ± 0.056	0.5112		
R17	Chr07MM2673-RM21871	TGW (g)	25.64 ± 0.351	25.04 ± 0.381	0.0694		
		GL (mm)	9.113 ± 0.054	9.110 ± 0.062	0.8963		
		GW (mm)	2.530 ± 0.026	2.524 ± 0.033	0.5378		
		RLW	3.618 ± 0.028	3.626 ± 0.050	0.5416		
R18	Chr07MM2673-SSR-9	TGW (g)	26.27 ± 0.365	26.08 ± 0.447	0.2481		
		GL (mm)	9.077 ± 0.058	9.090 ± 0.060	0.5687		
		GW (mm)	2.528 ± 0.019	2.518 ± 0.023	0.2132		
		RLW	3.606 ± 0.035	3.626 ± 0.036	0.1482		
R19	INDEL-2-Chr07MM2954	TGW (g)	23.12 ± 0.578	22.80 ± 0.512	0.0918		
		GL (mm)	8.550 ± 0.068	8.529 ± 0.066	0.3695		
		GW (mm)	2.537 ± 0.031	2.524 ± 0.026	0.1766		
		RLW	3.385 ± 0.047	3.394 ± 0.025	0.4264		
R20	C7-10-Chr07MM2954	TGW (g)	22.74 ± 0.746	22.46 ± 0.916	0.4744		
		GL (mm)	8.582 ± 0.082	8.609 ± 0.077	0.4788		
		GW (mm)	2.544 ± 0.040	2.519 ± 0.047	0.2436		
		RLW	3.391 ± 0.041	3.435 ± 0.066	0.0924		
R21	XP7-28-RM21891	TGW (g)	24.42 ± 0.519	24.51 ± 0.464	0.5903		
		GL (mm)	9.298 ± 0.115	9.199 ± 0.110	0.0124	−0.050	8.32
		GW (mm)	2.503 ± 0.028	2.516 ± 0.030	0.2037		
		RLW	3.734 ± 0.040	3.674 ± 0.067	0.0037	−0.030	19.21
R22	Chr07MM2954-RM21891	TGW (g)	23.62 ± 0.619	23.38 ± 0.519	0.2168		
		GL (mm)	8.677 ± 0.050	8.572 ± 0.095	<0.0001	−0.053	20.81
		GW (mm)	2.497 ± 0.029	2.493 ± 0.054	0.7792		
		RLW	3.489 ± 0.030	3.453 ± 0.054	0.0125	−0.018	6.62
R23	RM21866-RM21891	TGW (g)	27.10 ± 0.386	25.92 ± 0.383	<0.0001	−0.558	53.04
		GL (mm)	9.406 ± 0.084	9.223 ± 0.080	<0.0001	−0.087	42.21
		GW (mm)	2.566 ± 0.026	2.536 ± 0.040	0.0062	−0.011	13.18
		RLW	3.688 ± 0.032	3.655 ± 0.043	0.0122	−0.017	13.15
R24	C7-10-RM21891	TGW (g)	24.27 ± 0.464	23.26 ± 0.675	<0.0001	−0.505	33.52
		GL (mm)	8.699 ± 0.048	8.570 ± 0.087	<0.0001	−0.065	30.24
		GW (mm)	2.558 ± 0.036	2.519 ± 0.032	0.0027	−0.020	21.00
		RLW	3.416 ± 0.051	3.419 ± 0.028	0.8407		
R25	Chr07MM2870-RM21891	TGW (g)	26.40 ± 0.750	25.39 ± 0.356	0.0002	−0.504	34.58
		GL (mm)	9.276 ± 0.105	9.086 ± 0.066	<0.0001	−0.095	41.04
		GW (mm)	2.560 ± 0.227	2.520 ± 0.029	0.0005	−0.020	23.75
		RLW	3.640 ± 0.031	3.622 ± 0.031	0.1229		

*^a^TGW, thousand grain weight (g); GL, grain length (mm); GW, grain width (mm); RLW, the ratio of grain length to width.*

*^b^NIL^XLJ^ and NIL^BG1^ are homozygous genotypes from XLJ and BG1 in the segregating region, respectively.*

*^c^A, additive effect, measured as the genetic effect when an XLJ allele is replaced by a BG1 allele.*

*^d^R^2^, proportion of phenotypic variance explained by the QTL.*

Combined with the separated graphical genotypes of R15 and R21 populations, the *qTGW7.2* was dissected into two separate QTLs (Figure 1D). The first QTL, the *qTGW7.2a*, had considerable effects on TGW and GW within a 53.96 kb region spanning XP7-12 to XP7-16. The second QTL, the *qTGW7.2b*, was located between Chr07MM2985 and RM21891, a 52.71 kb interval, and had affected the GL and RLW but had little effect on the TGW.

Three NIL populations (N1-N3) derived from the R15, R16, and R21 populations were developed to validate the function of the *qTGW7.2a* and the *qTGW7.2b* (Supplementary Table 2). Meanwhile, two progeny populations, R26 and R27, derived from two recombinants only containing a *qTGW7.2a*, were used in this study (Supplementary Figure 2). In the N1 population, significant genotypic effects were detected for TGW and GW, which was coincident with R26 and R27 populations. Highly significant genotypic effects were detected for GL, GW, and RLW in the N3 population. There was no significant difference in the N2 population (Supplementary Table 2).

As described above, the *qGS7.1* was dissected into three independent QTLs. Primarily, the *qTGW7.1* was located in a region flanked by RM21772 and RM21858. The XLJ allele increased the TGW by.018g and.265g, and also the GW by.018mm and.031mm (Figure 1C and Table 2). The *qTGW7.2a* mainly controls TGW through regulating GW with enhancing alleles derived from XLJ. Simultaneously, the *qTGW7.2b* has affected the GL and GW in opposite ways with no significant effect on the TGW (Supplementary Figure 3). For further analysis, the *qTGW7.2a* was selected for its stable function and considerable effect.

### Fine-Mapping *qTGW7.2a* Into a 21.10-kb Region

For further mapping of *qTGW7.2a*, we constructed a BC_4_F_11_ population consisting of 6,128 individuals. Two recombinants in the RM21871-XP7-23 interval were utilized to develop two progeny populations, R28 and R29. Highly significant phenotypic effects were detected in TGW and GW in the R28 population. The additive effects were 0.213 g and 0.013 mm, having *R*^2^ of 11.29 and 9.70%, respectively. There were no significant differences in the R29 population (Table 4). According to the mapping results of the BC_4_F_12_ population, we mapped the *qTGW7.2a* to the 21.10 kb interval between Chr07MM2954 and XP7-16 (Figure 1E).

**TABLE 4:** Fine-mapping of *qTGW7.2a* using two residual heterozygous populations in BC_4_F_12_.

Name	Marker interval	Trait*^a^*	Phenotype (mean ± SD)*^b^*		*P*	*A^c^*	*R*^2^ (%)*^d^*
			NIL*^XLJ^*	NIL*^BG1^*			
R28	XP7-12-XP7-17	TGW (g)	25.42 ± 0.280	24.93 ± 0.398	<0.0001	–0.270	24.51
		GL (mm)	9.323 ± 0.072	9.293 ± 0.067	0.1920		
		GW (mm)	2.508 ± 0.026	2.483 ± 0.026	0.0050	–0.012	20.72
		RLW	3.734 ± 0.040	3.759 ± 0.030	0.0367	0.012	18.97
R29	XP7-12-Chr07MM2954	TGW (g)	25.10 ± 0.481	24.92 ± 0.510	0.3445		
		GL (mm)	9.173 ± 0.103	9.225 ± 0.101	0.1949		
		GW (mm)	2.475 ± 0.038	2.466 ± 0.037	0.5118		
		RLW	3.721 ± 0.037	3.757 ± 0.025	0.0890		

*^a^TGW, thousand grain weight (g); GL, grain length (mm); GW, grain width (mm); RLW, the ratio of grain length to width.*

*^b^NIL^XLJ^ and NIL^BG1^ are homozygous genotypes from XLJ and BG1 in the segregating region, respectively.*

*^c^A, additive effect, measured as the genetic effect when an XLJ allele is replaced by a BG1 allele.*

*^d^R^2^, proportion of phenotypic variance explained by the QTL.*

**FIGURE 2:**
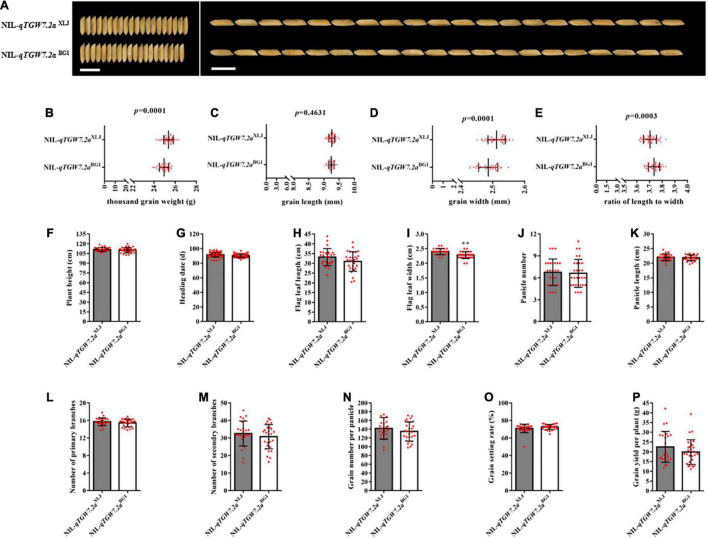
*qTGW7.2a* regulates grain width and weight. **(A)** Grain phenotypes of rice NIL plants. Bar = 1 cm. **(B)** Comparison of thousand grain weight. **(C)** Comparison of grain length. **(D)** Comparison of grain width. **(E)** Comparison of the ratio of grain length to width. **(F)** Comparison of plant height. **(G)** Comparison of heading date. **(H)** Comparison of flag leaf length. **(I)** Comparison of flag leaf width. **(J)** Comparison of panicle number. **(K)** Comparison of panicle length. **(L)** Comparison of number of primary branches. **(M)** Comparison of secondary branches. **(N)** Comparison of grain number per panicle. **(O)** Comparison of grain setting rate. **(P)** Comparison of grain yield per plant. Data are given as mean ± SD. Student’s *t*-test was used to generate *P* value; ^**^P < 0.01; red dot, the number of plants for traits measurement.

**FIGURE 3:**
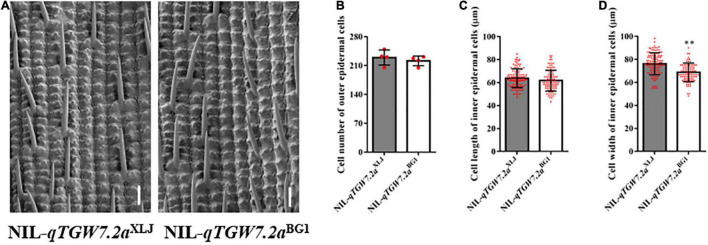
Scanning electron microscopic observation and analysis of the glume. **(A)** Scanning electron micrograph of the outer glume epidermal cells between NIL-*qTGW7.2a^XLJ^* and NIL-*qTGW7.2a^BG1^*. Bar = 100 μm. **(B)** Cell number of outer epidermal cells. **(C)** Cell length of outer epidermal cells. **(D)** Cell width of outer epidermal cells. Data are given as mean ± SD. Student’s *t*-test was used to generate *P* value; ^**^P < 0.01. Red dot, the number of grains used to calculate cell number in **(B)**, and cell numbers to measure cell length **(C)** and cell width **(D)**.

The *qTGW7.2a* increased the TGW and GW with the enhancing allele from XLJ as compared to grain size and weight between NIL-*qTGW7.2a^XLJ^* and NIL-*qTGW7.2a^BG1^* (Figures 2A–E), which were derived from the R28 population. Additionally, compared with NIL-*qTGW7.2a^BG1^*, besides the flag leaf width, the NIL-*qTGW7.2a^XLJ^* showed comparable effects in the heading date, plant height, panicle number and length, grain number per panicle, and other agronomic traits (Figures 2F–P). Grain size is restricted by the size of the spikelet hull in rice, which is determined by both cell proliferation and expansion. Therefore, we compared the cell number and the cell size of the outer glume epidermal cells between NIL-*qTGW7.2a^XLJ^* and NIL-*qTGW7.2a^BG1^* (Figure 3A). There was no significant difference in cell number or cell length between NIL-*qTGW7.2a^XLJ^* and NIL-*qTGW7.2a^BG1^* (Figures 3B,C). However, the cell width of NIL-*qTGW7.2a^XLJ^* was significantly greater than NIL-*qTGW7.2a^BG1^* (Figure 3D). These findings suggest that the grain size increase in NIL-*qTGW7.2a^XLJ^* is predominantly due to the cell width expansion.

### Candidate Genes of *qTGW7.2a* and *qTGW7.2b*

There are four open reading frames (*ORFs*) located in the region spanning *qTGW7.2a*. The *LOC_Os07g39470* encodes a rice GRAS family protein, CIGR2, which suppresses cell death in rice inoculated with a rice blast *via* activation of a Heat Shock Transcription Factor, OsHsf23 (Tanabe et al., 2016). The *LOC_Os07g39480* encodes WRKY78, a transcriptional factor that is involved in regulating plant height and seed size (Zhang et al., 2011). Lastly, the *LOC_Os07g39490* and *LOC_Os07g39500* are unknown functional proteins (Supplementary Table 3).

Sequences of the genome sequence in four genes between the NIL-*qTGW7.2a^XLJ^* and NIL-*qTGW7.2a^BG1^* were compared. Mutations were found at 61, 47, 43, and 77 sites of the four genes, respectively (Supplementary Tables 4–7). Two synonymous SNPs were detected in *LOC_Os07g39470*, indicating that there were no differences between the two alleles. For *LOC_Os07g39480*, there were four polymorphic sites, three of which were synonymous and one 3-bp deletion in the XLJ allele, resulting in a serine deletion. For *LOC_Os07g39490*, three SNPs include one synonymous and two non-synonymous were detected between two NILs, resulting in two amino acids substituted; especially, a 2-bp deletion in NIL-*qTGW7.2a^BG1^* led to the terminal 62 residues were truncated. Finally, there were 16 SNP variations in *LOC_Os07g39500* between two NILs, including twelve non-synonymous mutations and a premature stop codon at T784C in the BG1 allele. However, there were three fragment deletions/insertions in the promoter and one at the 5′-UTR region of *LOC_Os07g39470*. One fragment deletions/insertion of *LOC_Os07g39480* was at its promoter (Supplementary Tables 6, 7).

**FIGURE 4:**
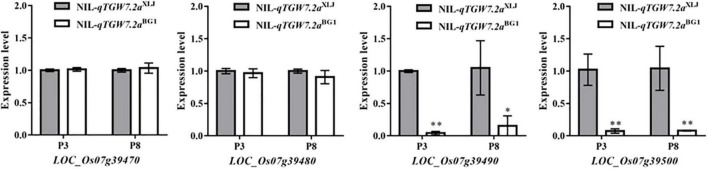
The transcript levels of annotated genes between NIL-*qTGW7.2a^XLJ^* and NIL-*qTGW7.2a^BG1^*. The experiment was performed using panicles of 1 ≤ P < 3 cm (P3) and 5 ≤ P < 8 cm (P8) collected from NIL-*qTGW7.2a^XLJ^* and NIL-*qTGW7.2a^BG1^*. Data are given as mean ± SD. Student’s *t*-test was used to generate *P* value; *P < 0.05; ^**^P < 0.01.

Subsequently, the expression levels of four candidates in the panicles of NIL-*qTGW7.2a^XLJ^* and NIL-*qTGW7.2a^BG1^* were analyzed (Figure 4). The expression levels of the *LOC_Os07g39490* and the *LOC_Os07g39500* were significantly higher in NIL-*qTGW7.2a^XLJ^* than in NIL-*qTGW7.2a^BG1^*, while there were no significant differences between the two NILs in the *LOC_Os07g39470* and the *LOC_Os07g39480*.

According to the Gramene Database^1^, there are eight candidate genes in the *qTGW7.2b* region (Supplementary Table 8). One of them encodes proteins with known functional domains. The *LOC_Os07g39750* encodes acetylcholinesterase relating to the positive regulation of gravitropic response in rice seedlings (Yamamoto et al., 2015). Four of them encode putative functional proteins, including *LOC_Os07g39740*, which encodes a GDSL-like lipase/acyl hydrolase, *LOC_Os07g39780* for a SUMO-activating enzyme subunit 2, *LOC_Os07g39800* for a transcription repressor HOTR, and *LOC_Os07g39810*, which encodes a protein that belongs to the lipase class 3 family. The remaining annotated genes *LOC_Os07g39760*, *LOC_Os07g39770*, and *LOC_Os07g39790* are for unknown functional proteins.

Sequence comparisons of the eight annotated genes between NIL-*qTGW7.2b^XLJ^* and NIL-*qTGW7.2b^BG1^* were performed. Thirty-eight variants were found of the *LOC_Os07g39810* (Supplementary Table 9). No variants were detected in the sequences of *LOC_Os07g39760* and *LOC_Os07g39770* and in the 2-6 sites of the other five genes (Supplementary Table 10), but few of them occurred in the coding sequence (CDS). For *LOC_Os07g39790*, three mutations were identified, including the C2088G and the T2233C synonymous substitution, as well as a three-nucleotide insertion in NIL-*qTGW7.2b^XLJ^*. For the other four genes, most SNPs were detected in the promoter and terminator regions.

## Discussion

Grain size is jointly regulated by large-effect and small-effect QTLs. Remarkable progress has been achieved by the discovery of large-effect QTLs affecting the yield and quality in recent years. Notably, rarely have small-effect QTLs been cloned in rice (Chan et al., 2020; Du et al., 2021). It is recognized that the complex traits, especially as grain yield and its component traits, were controlled by many genes. However, in recent years, the number of the cloned large-effect QTLs is decreasing. Small-effect QTL does not have the same effect as the large-effect QTL, but compared with the large-effect QTL, the small-effect QTL usually affects a single trait rather than the multiple agronomic traits or, even, the adverse changes. So, the small-effect QTLs could be selected and be used to fine-tune the function of a large-effect QTL, thereby making the varieties fitter to the market demand. Aggregation of small-effect QTLs with the same genetic direction may even show greater phenotypic changes. Moreover, molecular characterization of these small-effect QTLs, which have a direct and consistent influence on grain yield, would be beneficial to establish a regulatory network for rice grain development. These indicate that if we pay more attention to the small-effect QTLs, it is possible to detect some dominant alleles that have not been selected, and they will have more potential applications in breeding.

In the present study, the *qGS7.1* has increased the TGW, GL, and GW by 3.17, 0.27, and 0.86%, respectively, with the enhancing allele derived from XLJ (Table 1). Then, the *qGS7.1* is dissected into three closely linked QTLs, *qTGW7.1*, *qTGW7.2a*, and *qTGW7.2b*. The latter two small-effect QTLs, regulating grain size and grain shape, were identified and fine-mapped. Grain size and grain shape are both determined by grain length and width. While grain size is the main factor affecting the grain weight, grain shape mainly influences grain preference and may not be related to grain weight (Dong et al., 2018). *qTGW7.2a* was limited between Chr07MM2954 and XP7-16 with a 21.10 kb interval, affecting grain width and weight. *qTGW7.2b*, which inversely affects gra in length and width, was mapped into the 52.71 kb region between Chr07MM2985 and RM21891.

Small-effect QTLs were susceptible to environmental and genetic background. However, the effects of a genetic context could be counteracted under a highly homozygous genetic context. All the populations used in this study were derived from a single plant of high generation BC_4_F_6_ populations with the same background and were cultivated in Fuyang and Lingshui following the chronological order. The *qTGW7.2a* could be detected in both environments, but the effects on TGW and GW were not stable. The additive effects on TGW and GW increased by XLJ allele were in the range of 0.213 to 0.604 g and 0.013 to 0.034 mm in the two environments, respectively (Tables 3, 4 and Supplementary Table 2). Especially for *qTGW7.2b*, in R21 and R22 populations, the *qTGW7.2b* regulates the grain length and has little influence on the grain width and grain weight. Despite this, in the N3 population, the *qTGW7.2b* was detected affecting the grain length and grain width with opposite allelic directions and had little effect on grain weight (Table 3 and Supplementary Table 2). These results suggested that small-effect QTLs could be steadily detected using the residual heterozygous method, but the effects of QTL could be affected by environmental interaction.

Among QTLs with large effects, *GW2*, *GS5*, *GW5/GSE5*, and *GW6* are those which regulate grain weight through controlling grain width (Song et al., 2007; Li et al., 2011; Xu et al., 2015; Duan et al., 2017; Liu et al., 2017; Shi et al., 2020). In our study, the *qTGW7.2a* has increased the grain width and weight but did not influence the other agronomic traits. These suggest that the *qTGW7.2a* could be used for yield improvement.

For large-effect QTLs, the following have similar effects with *qTGW7.2b* on grain length and width that regulate grain shape: *GL7*/*GW7*, *GW8*, and *GS9* (Wang et al., 2012; Wang S. K. et al., 2015; Wang Y. X. et al., 2015; Zhao et al., 2018). In the XLJ allele, the *qTGW7.2b* increased the grain length but decreased the grain width. This result has little effect on grain weight and, therefore, indicated that the *qTGW7.2b* could be used to fine-tune the grain size.

Four annotated genes were found in the 21.1 kb interval covering the *qTGW7.2a*. Firstly, the *LOC_Os07g39470* encodes CIGR2 belonging to the rice GRAS family, and members of this family encode transcriptional regulators with functions in a wide range of signaling mechanisms, such as growth and development, hormone signaling, and plant defense (Tanabe et al., 2016). However, there were only two synonymous SNPs between the *CIGR2* alleles. Secondly, the *LOC_Os07g39480* encodes a transcriptional factor, the WRKY78, which was involved in regulating the plant height and seed size. Knocking down of the *WRKY78* led to a semi-dwarf and small seed phenotype by reducing the cell length (Zhang et al., 2011). However, in addition to the three SNPs showing synonymous mutation, there was just one serine deletion in the CDS of NIL-*qTGW7.2a^XLJ^*. The expression level of the *WRKY78* was comparable between the two NILs. Combined with the fragment deletions/insertion of the *LOC_Os07g39470* and the *LOC_Os07g39480*, these results may be due to how the deletions/insertion at the promoter region were not the cis-acting element and does not affect their mRNA expression level. Previous studies showed that the coding region introduced a premature stop codon resulting in premature termination of translation could influence grain size, such as *GW2*, *GS3*, *qLGY3*/*GW3p6*, *WTG1*, *OsMAPK6*, *TGW6*, and *GL6* (Fan et al., 2006; Song et al., 2007; Ishimaru et al., 2013; Liu S. Y. et al., 2015; Huang et al., 2017; Liu et al., 2018; Wang A. H. et al., 2019; Wang C. S. et al., 2019). In our study, the *LOC_Os07g39490* and the *LOC_Os07g39500* encode hypothetical proteins. In its coding region, a non-synonymous mutation existed as a premature stop codon and prevents the transcription of a mature protein in NIL-*qTGW7.2a^BG1^*. Therefore, more studies in the gene editing, such as CRISPR/Cas9-targeted mutagenesis and gene overexpression, need to be done to confirm the gene for the *qTGW7.2a*.

The 52.71 kb region surrounding the *qTGW7.2b* contained eight annotated genes. For two annotated genes, *LOC_Os07g39760* and *LOC_Os07g39770*, no variants between NIL-*qTGW7.2b^XLJ^* and NIL-*qTGW7.2b^BG1^* were detected. For four other annotated genes, *LOC_Os07g39740*, *LOC_Os07g39750*, *LOC_Os07g39780*, and *LOC_Os07g39800*, only a few SNPs were detected at the beginning of promoter regions and the terminator regions, and no variants were detected in the coding domain sequence. For the *LOC_Os07g39780*, there were two synonymous mutations and a Ser insertion detected in the NIL-*qTGW7.2b^XLJ^*. For the remaining annotated gene, the *LOC_Os07g39810*, which encodes an expression protein, three long fragment insertions in the promoter region, and an insertion of nine nucleotides or three amino acids were detected in the NIL-*qTGW7.2b^XLJ^*. Work is underway to test whether *LOC_Os07g39780* or *LOC_Os07g39810* is the gene underlying the QTL *qTGW7.2b* for grain shape.

## Conclusion

Two small-effect QTLs for grain size and grain shape, *qTGW7.2a* and *qTGW7.2b*, were fine-mapped in this study. *qTGW7.2a* was limited to a 21.10 kb region containing four genes. This QTL regulates grain width and weight, which has potential for yield improvement. The *qTGW7.2b*, which inversely regulates grain length and width, was within a 52.71 kb interval. This QTL has potential for fine-tuning the grain shape and the grain appearance. These results provide a basis for QTL cloning and offer new resources for the yield and quality improvement.

## Data Availability Statement

The original contributions presented in the study are included in the article/Supplementary Material, further inquiries can be directed to the corresponding authors.

## Author Contributions

PX performed most of the experiments, analyzed the data, and wrote the manuscript. Y-YC contributed to sequencing and constructed the populations. X-XW, B-FW, and Q-QY analyzed the data collection the phenotypes and revised the manuscript. KG and Y-WK conducted the field trials. L-PS, PY, L-YC, Y-XZ, X-DZ, and S-HC designed the experiments, supervised and completed the writing, and reviewed the manuscript. All authors read and approved the final manuscript.

## Conflict of Interest

The authors declare that the research was conducted in the absence of any commercial or financial relationships that could be construed as a potential conflict of interest.

## Publisher’s Note

All claims expressed in this article are solely those of the authors and do not necessarily represent those of their affiliated organizations, or those of the publisher, the editors and the reviewers. Any product that may be evaluated in this article, or claim that may be made by its manufacturer, is not guaranteed or endorsed by the publisher.

## Footnotes

1
https://www.gramene.org/
