# Supplementary material for: Dissection of Closely Linked Quantitative Trait Locis Controlling Grain Size in Rice
Source: Front Plant Sci. 2022 Jan 20;12:804444. doi: 10.3389/fpls.2021.804444 (PMC8810522; doi:10.3389/fpls.2021.804444)
Supplement: Supplementary file 1 [file Data_Sheet_1.PDF]

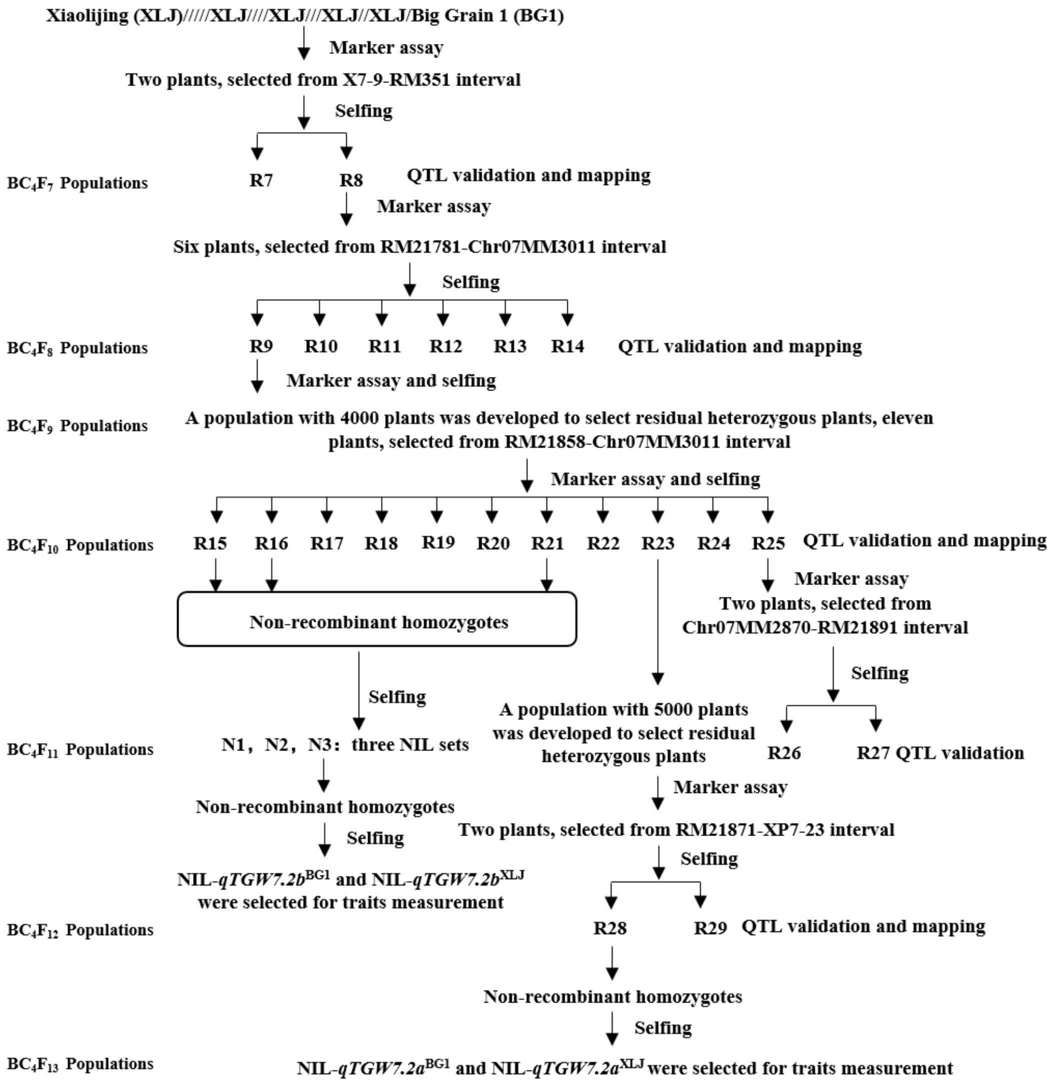

FIGURE S1 | Population development in this study.

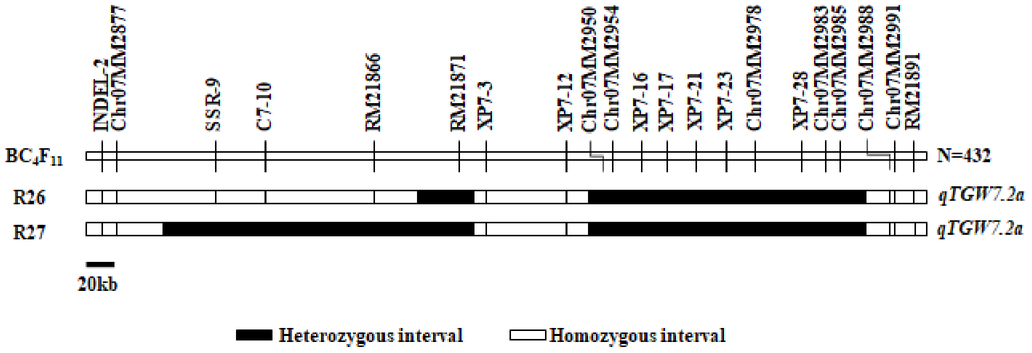

FIGURE S2 | Validation of *qTGW7.2a* in BC4F11.

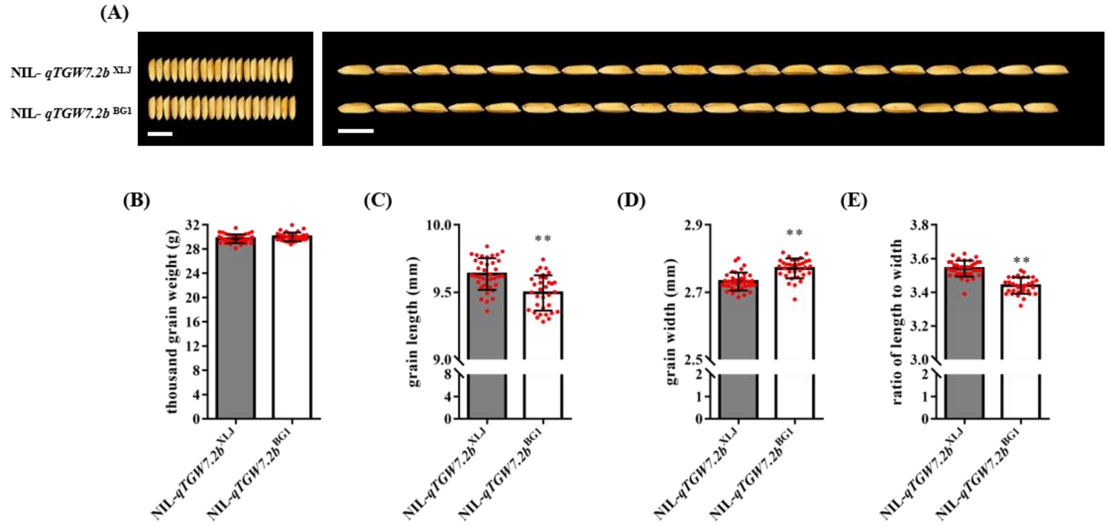

FIGURE S3 | *qTGW7.2b* regulates grain length and width. **(A)** Grain phenotypes of rice NIL plants. Bar, 1 cm. **(B)** Comparison of thousand grain weight. **(C)** Comparison of grain length. **(D)** Comparison of grain width. **(E)** Comparison of the ratio of grain length to width. Data are given as mean ± SD. Student's *t*-test was used to generate *P* value; *, $P < 0.05$; **, $P < 0.01$; red dot, the number of plants for traits measurement.

TABLE S1 | Primers used in this study.

Marker	Forward primer (5'→3')	Reverse primer (5'→3')	Function
RM21758	GATTCTAATCACGCCTCCCATCC	CGTCCTCGTCTCGAGTAAGTTGC	QTL-mapping
RM21772	TGCTCCTCGTCCTAAACAGTTGG	CCGTGGCGAAATACTTGATGG	
Chr07MM2526	TTCCATTTAAGTGCGGTGTG	CCATTGGAGGAAGGAGATGA	
Chr07MM2577	ACCTCCGACCTGCTGTACC	CACGTAAGCAGCGATCAAGA	
InDel7-14	TCTCAGTGACGCAAAGTTTCCT	ACAGCACGTACGCATGAAGG	
Chr07MM2673	GGGGGAAGAAGAGTAGTGGG	CAAAGGAAAATTGCATGCTG	
RM21858	CACAAGACAGGCCAAACAAGAGC	CCTCCTCCAACCGCCTATTGC	
Chr07MM2870	CTCCTCCGCCCTTTTATCTC	TCTTCTCCTCCCTCCTCCTC	
INDEL-2	CAGCACGCAAAATGAGAC	CCAAACTGCCAAACAACA	
Chr07MM2877	AGGTTTCCTGTGGTTCTGTGA	TCACCCCAAGCTACCAAAAC	
SSR-9	GTGGCCCAAACCCTAACCTA	AAACTCAGATGCAAGCAGCAC	
C7-10	TCCGGCGAGAAAATAAGTGT	TTCGTTAATCCTCACGCAGA	
RM21866	CTCATCCTGCGAGTTATCCAACC	CGGTCTGATTGAAGGTGATGTAC	
RM21871	ATCCCGATGCCTCTCTGACTCC	CGAAGAGGAAGGGAAGGGAAGG	
XP7-3	GCGGAGACATCATGGGCTTA	GTACTCCGGGACATACACCA	
XP7-12	ATTGGATGGAGCTAAGCCGA	TTGACAAGAGGGTGACTGGT	
Chr07MM2950	GAAGGAGATCTTGGACGCTG	GTTCTAAGATCCTGGCGTGC	
Chr07MM2954	CATGCGAATGCCGTAATCTA	CTCCTGTCCAGCTAACCAGC	
XP7-13	AGCACCTTTAGAGTGGATTATC	AGATGTCGTGTCATTTGAAGAG	
XP7-16	TAGAGGTGGACCCATCAGTG	GAACGGTTAAATGTTGGGCA	
XP7-17	ATCCCAAAGGTACTGTTGTCC	TGTTGTAAACTACACCCTCTCA	
XP7-21	GAGTGCGGAAAACAAGTGAC	TTCTGACAAGGTTCATGCGT	
XP7-23	ATTCAGCCCCTGTTAGAGGA	ACAAAATATTGCACCTCCCGT	
Chr07MM2978	AGTAGAACACCGACGATGGG	GTTTTTATGGCACGAAGGGA	
XP7-28	TCAAACGGTCTTGCCTGGTC	GGCTGAAAGCTTGAAAAGGAAC	
Chr07MM2983	GATGGTGGAGGAGAGAAGGG	AAACAAATCGAATTCGCACC	
Chr07MM2985	CAGCTACTCAAATGGTTATAAAGAA	TTCCTTGTTGTTTGCATGTGA	
Chr07MM2988	AATTACCTGGTGCTCCATCG	AGCTGTGTTCTCTCCTGCGT	
Chr07MM2991	CGTTGAAGAGGTGGAACTCG	GTCGGAGAACGTCTGCCTCT	
RM21891	TGCTTGCTTCGATCTGATCTCC	TCCAGTTGTGACTTGTGAAGAAG	
Chr07MM3011	TTCCTAAACAAATCCGCCAC	TACTCCTCTCAACCGCGTCT	
LOC_Os07g3947	AGAGCTACTGATTGCCTGTGC	TGAAATGCCAGAGGAGGCAAG	qRT-PCR
LOC_Os07g3948	TCTCCGACAACTGGTACTCTGA	TGGGTGCTACCAGATGTTTTAT	
LOC_Os07g3949	ATGGCTCATCTGATACACCAAGT	CATGCCCAGTGGATTCGGATA	
LOC_Os07g3950	CGGCGCTGGAGAAGCTAGAA	GTGGGGGTAGAGCCGTAGAG	
UBQ10	TGGTCAGTAATCAGCCAGTTTGG	GCACCACAAATACTTGACGAACA	

TABLE S2 | Validation of *qTGW7.2a* and *qTGW7.2b* using three near isogenic lines and two residual heterozygous populations in BC_4F_{11}.

Name	Marker interval	Trait [a]	Phenotype (mean±SD) [b]		P	A [c]	R^2 (%) [d]
			NIL^{XLJ}	NIL^{BG1}			
N1	Chr07MM2673-Chr07MM2978	TGW(g)	29.78±0.636	28.94±0.623	<0.0001	-0.421	31.59
		GL(mm)	9.212±0.092	9.194±0.086	0.3376		
		GW(mm)	2.807±0.027	2.766±0.024	<0.0001	-0.021	40.05
		RLW	3.293±0.040	3.336±0.039	<0.0001	0.021	23.22
N2	Chr07MM2673-Chr07MM2954	TGW(g)	29.80±0.592	29.93±0.585	0.3278		
		GL(mm)	9.393±0.093	9.409±0.099	0.4522		
		GW(mm)	2.715±0.029	2.720±0.032	0.4564		
		RLW	3.470±0.042	3.471±0.053	0.9676		
N3	XP7-28-RM21891	TGW(g)	29.72±0.716	30.00±0.705	0.1023		
		GL(mm)	9.635±0.115	9.495±0.128	<0.0001	-0.070	25.78
		GW(mm)	2.732±0.026	2.771±0.029	<0.0001	0.020	34.30
		RLW	3.540±0.047	3.438±0.047	<0.0001	-0.051	54.26
R26	RM21866-Chr07MM2985	TGW(g)	29.05±1.002	28.30±0.830	<0.0001	-0.379	9.07
		GL(mm)	9.558±0.177	9.575±0.130	0.5985		
		GW(mm)	2.736±0.056	2.703±0.053	0.0039	-0.016	4.54
		RLW	3.507±0.013	3.556±0.084	0.0025	0.025	5.67
R27	Chr07MM2673-Chr07MM2985	TGW(g)	30.26±1.113	29.68±0.968	0.0043	-0.291	3.73
		GL(mm)	9.649±0.189	9.650±0.218	0.9826		
		GW(mm)	2.727±0.059	2.681±0.060	<0.0001	-0.023	5.82
		RLW	3.554±0.081	3.616±0.082	0.0001	0.031	6.47

a TGW, thousand grain weight (g); GL, grain length (mm); GW, grain width (mm); RLW, the ratio of grain length to width.

b NIL^{XLJ} and NIL^{BG1} are homozygous genotypes from XLJ and BG1 in the segregating region, respectively.

c *A*, additive effect, measured as the genetic effect when an XLJ allele is replaced by a BG1 allele.

d R^2, proportion of phenotypic variance explained by the QTL.

TABLE S3 | Predicated candidate genes of *qTGW7.2a*.

ORF	Gene function
LOC_Os07g39470	GRAS family protein, gibberellin response modulator protein
LOC_Os07g39480	WRKY transcription factor 78
LOC_Os07g39490	Expressed protein
LOC_Os07g39500	Expressed protein

TABLE S4 | Sequence comparison of *LOC_Os07g39470* between the NIL-*qTGW7.2a*^{XLJ} and NIL-*qTGW7.2a*^{BG1}.

Construction	Genome position	NIL-*qTGW7.2a* ^{XLJ}	NIL-*qTGW7.2a* ^{BG1}	Variation
**Promoter region (1930bp)**	52	C	T	SNP
	73	C	T	SNP
	80	–	C	SNP
	83	–	A	SNP
	98	C	G	SNP
	99	T	C	SNP
	200-201	AA	– –	InDel
	288	C	T	SNP
	304	A	G	SNP
	305-307	ACC	– – –	InDel
	369	T	A	SNP
	393-394	––	CA	InDel
	395	G	A	SNP
	405	G	A	SNP
	534	C	T	SNP
	544	T	C	SNP
	550	G	A	SNP
	731	G	T	SNP
	834	C	T	SNP
	854	A	G	SNP
	934	C	T	SNP
	995	A	C	SNP
	996-1052	TTA*19	————————	InDel
	1073	T	C	SNP
	1088	G	A	SNP
	1173	T	C	SNP
	1189	T	G	SNP
	1237	A	G	SNP
	1276	A	G	SNP
	1292	C	T	SNP
	1328	A	G	SNP
	1406-1422	————————	CTTTAATTTTTATAATA	InDel
	1433	A	G	SNP
	1462	A	G	SNP
	1468	T	C	SNP
	1502	C	A	SNP
	1528-1530	–––	CTA	InDel
	1532-1533	AT	GA	SNP
	1542	A	G	SNP
	1549	A	G	SNP
	1557	T	C	SNP
	1561	T	C	SNP
	1616	A	G	SNP
	1684	C	T	SNP
	1690-1701	AGC*4	————————	InDel
	1886	A	–	SNP
	1892	–	C	SNP
**5'-UTR**	1994-2005	————————	CACCACCACCAC	InDel
**Intron**	2427-2431	GTTAA	–––––	InDel
	2566	C	G	SNP
	2594	A	G	SNP

	3046	A	T	SNP
	3066	T	C	SNP
**5'-UTR**	3446	A	G	SNP
**CDS**	3864	GAT (Asp)	GAC (Asp)	Synonymous mutation
	4701	GAA (Glu)	GAG (Glu)	Synonymous mutation
**3'-UTR**	5182	C	T	SNP
	5206	C	G	SNP
**Terminator region (862bp)**	5598	A	T	SNP
	5936	C	T	SNP
	6208-6210	———	GGA	InDel

The horizontal line indicates base deletion; the red word represent mutations in the coding domain sequence.

TABLE S5 | Sequence comparison of *LOC_Os07g39480* between the NIL-*qTGW7.2a*^{XLJ} and NIL-*qTGW7.2a*^{BG1}.

Construction	Genome position	NIL-*qTGW7.2a* ^{XLJ}	NIL-*qTGW7.2a* ^{BG1}	Variation
Promoter region (1346bp)	150	T	G	SNP
	169	G	C	SNP
	186	C	A	SNP
	301	T	G	SNP
	314	G	A	SNP
	360	G	T	SNP
	361	−	T	SNP
	363	T	A	SNP
	367	T	C	SNP
	368	G	A	SNP
	369	T	C	SNP
	372	G	C	SNP
	374	T	A	SNP
	375	T	A	SNP
	380-390	———————	CCAGATTATAA	InDel
	393	C	A	SNP
	396	C	A	SNP
	400	C	A	SNP
	401-403	− − −	CTC	InDel
	422	C	T	SNP
	440	A	T	SNP
	538	G	A	SNP
	624	G	A	SNP
	692	A	T	SNP
	959	T	G	SNP
	960	G	A	SNP
	987	G	A	SNP
	1004	G	C	SNP
	1164	T	G	SNP
Exon1	1507	GCA (Ala)	GCG (Ala)	Synonymous mutation
Intron1	1852	T	G	SNP
	1906	T	C	SNP
	2287	T	C	SNP
Exon2	2837	GAG (Glu)	GAA (Glu)	Synonymous mutation
Intron2	3290	C	T	SNP
	3362	C	T	SNP
Exon3	4012	ATT (Ile)	GTT (Ile)	Synonymous mutation
Intron3	4507	−	C	SNP
	4776	G	T	SNP
	4992	T	G	SNP
	5021	A	G	SNP
Exon6	6166-6168	TCCTCA (Ser Ser)	TC−−−A (Ser)	Non-synonymous mutation
3'-UTR	6437	A	G	SNP
	6775	A	T	SNP
Terminator region (1000bp)	7170	C	G	SNP
	7194	A	G	SNP
	7585	C	T	SNP

The horizontal line indicates base deletion; the red word represent mutations in the coding domain sequence.

TABLE S6 | Sequence comparison of *LOC_Os07g39490* between the NIL-*qTGW7.2a*^{XLJ} and NIL-*qTGW7.2a*^{BG1}.

Construction	Genome position	NIL-*qTGW7.2a* ^{XLJ}	NIL-*qTGW7.2a* ^{BG1}	Variation
Promoter region (1967bp)	26	C	A	SNP
	69	C	T	SNP
	451	A	–	SNP
	592	–	A	SNP
	593	A	G	SNP
	677	A	C	SNP
	786	A	G	SNP
	794-796	TAT	– – –	InDel
	851	T	C	SNP
	975	C	A	SNP
	992	C	G	SNP
	1002	T	C	SNP
	1034	T	C	SNP
	1118	A	G	SNP
	1154	C	T	SNP
	1166	C	T	SNP
	1354	A	G	SNP
	1356	–	G	SNP
	1457	A	G	SNP
	1536	G	–	SNP
	1539	C	T	SNP
	1561	G	A	SNP
	1672	A	G	SNP
	1682	T	C	SNP
	1736	T	C	SNP
	1780	–	A	SNP
	1793	T	C	SNP
	1794	A	G	SNP
	1808	C	T	SNP
	1822	G	A	SNP
	1850	C	T	SNP
	1862	C	T	SNP
	1866	T	C	SNP
	1935	G	A	SNP
	1951	C	T	SNP
Intron1	2228	G	A	SNP
	2266	C	G	SNP
	2297	T	C	SNP
Intron2	2446	G	A	SNP
Exon3	2510	AAT (Asn)	GAT (Asp)	Non-synonymous mutation
	2562	TCT (Ser)	TGT (Cys)	Non-synonymous mutation
	2567	AAT (Asn)	TAT (Tyr)	Non-synonymous mutation
	2570-2571	GAT (Asp) GA	—TGA	Premature stop

The horizontal line indicates base deletion; the red word represent mutations in the coding domain sequence.

TABLE S7 | Sequence comparison of *LOC_Os07g39500* between the NIL-*qTGW7.2a*^{XLJ} and NIL-*qTGW7.2a*^{BG1}.

Construction	Genome position	NIL-*qTGW7.2a* ^{XLJ}	NIL-*qTGW7.2a* ^{BG1}	variation
	19	G	A	SNP
	62	A	G	SNP
	70	A	G	SNP
	115	A	G	SNP
	129	A	G	SNP
	196	−	C	SNP
	219	T	C	SNP
	239	A	G	SNP
	262	C	T	SNP
	384	C	−	SNP
	390	C	T	SNP
	400	A	G	SNP
	415	T	C	SNP
	461	G	A	SNP
	493	C	T	SNP
	494	A	G	SNP
	501	A	G	SNP
	513	−	T	SNP
	522	A	T	SNP
	529	C	T	SNP
	538	C	T	SNP
	561	A	G	SNP
	565	T	C	SNP
	569	G	A	SNP
**Promoter region**	578	−	T	SNP
**(1672bp)**	644	C	G	SNP
	680	A	G	SNP
	693	−	T	SNP
	701-702	− −	AG	InDel
	711	−	T	SNP
	740	−	A	SNP
	853	A	G	SNP
	884	C	T	SNP
	922	A	G	SNP
	939	T	C	SNP
	965	T	C	SNP
	994	C	T	SNP
	996	A	C	SNP
	1002	A	G	SNP
	1015	T	C	SNP
	1050	A	G	SNP
	1058	C	T	SNP
	1117	G	A	SNP
	1400	T	G	SNP
	1402	T	G	SNP
	1480	T	C	SNP
	1529	C	T	SNP
	1531	A	G	SNP
	1656	C	G	SNP
	1667	T	G	SNP
	1680	GCG (Ala)	GTG (Val)	Non-synonymous mutation

Exon1	1688	ACG (Thr)	GCG (Ala)	Non-synonymous mutation
	1696	CCA (Pro)	CCG (Pro)	Synonymous mutation
	1699	ACT (Thr)	ACC (Thr)	Synonymous mutation
	1745	CGC (Arg)	TGC (Cys)	Non-synonymous mutation
	1833	CAA (Gln)	CGA (Arg)	Non-synonymous mutation
	1853	ATC (Ile)	GTC (Val)	Non-synonymous mutation
	1863	CCC (Pro)	CTC (Leu)	Non-synonymous mutation
	1866	GAC (Asp)	GGC (Gly)	Non-synonymous mutation
Intron1	1890	C	–	SNP
	1967	T	C	SNP
	1982	C	–	SNP
	1988	C	T	SNP
	2117-2118	– –	TT	InDel
	2142	A	G	SNP
	2174	T	G	SNP
	2196	T	G	SNP
	2201	A	G	SNP
	2441	T	C	SNP
	2465	–	T	SNP
Exon2	2667	AGC (Ser)	AAC (Phe)	Non-synonymous mutation
	2713	AAA (Phe)	AAG (Lys)	Non-synonymous mutation
	2749	GGG (Gly)	GGC (Gly)	Synonymous mutation
	2759	ATG (Met)	GTG (Val)	Non-synonymous mutation
	2892	AGA (Cys)	ATA (Ile)	Non-synonymous mutation
	2901	ATA (Ile)	ACA (Thr)	Non-synonymous mutation
	3059	CAG (Gln)	TAG (STOP)	Premature stop

The horizontal line indicates base deletion; the red word represent mutations in the coding domain sequence.

TABLE S8 | Predicated candidate genes of *qTGW7.2b*.

ORF	Gene function
LOC_Os07g39740	GDSL-like lipase/acyl hydrolase
LOC_Os07g39750	Acetylcholinesterase gene
LOC_Os07g39760	Expressed protein
LOC_Os07g39770	Expressed protein
LOC_Os07g39780	SUMO-activating enzyme subunit 2
LOC_Os07g39790	Expressed protein
LOC_Os07g39800	Transcription repressor HOTR
LOC_Os07g39810	Lipase class 3 family protein

TABLE S9 | Sequence comparison of *LOC_Os07g39810* between the NIL-*qTGW7.2b*^{XLJ} and NIL-*qTGW7.2b*^{BG1}.

Construction	Genome position	NIL-*qTGW7.2b* ^{XLJ}	NIL-*qTGW7.2b* ^{BG1}	Variation
**Promoter region (1911bp)**	19	G	A	SNP
	60	A	G	SNP
	186	C	T	SNP
	411	A	G	SNP
	492-494	AAT	– – –	InDel
	498	C	–	SNP
	523	A	C	SNP
	598	G	A	SNP
	636	G	A	SNP
	705	A	–	SNP
	729	A	–	SNP
	759	T	–	SNP
	815-821	CACCACT	————	InDel
	854	G	A	SNP
	1053	–	G	SNP
	1065	G	A	SNP
	1108	G	A	SNP
	1164	G	A	SNP
	1222	G	A	SNP
	1243	G	A	SNP
	1245	G	C	SNP
	1274	C	T	SNP
	1298-1319	AGAAAAAAAACTTGCTCTAGCA	————	InDel
	1503	G	A	SNP
	1803-1812	AGCTTAGCTT	————	InDel
**CDS**	2302	GCA (Ala)	GCG (Ala)	Synonymous mutation
	2482	GGA (Gly)	GGC (Gly)	Synonymous mutation
	2558-2566	GCCGCCGCC (Ala-Ala-Ala)	————	Deletion
**3'-UTR**	2851	A	C	SNP
	3028	A	C	SNP
**Terminator region (916bp)**	3206	A	T	SNP
	3363	A	G	SNP
	3417	G	A	SNP
	3486	C	G	SNP
	3651	T	A	SNP
	3738	T	A	SNP
	3849	T	A	SNP
	3959	G	A	SNP

The horizontal line indicates base deletion; the red word represent mutations in the coding domain sequence.

TABLE S10 | Sequence comparison of seven annotated genes between the NIL-*qTGW7.2b*^XLJ and NIL-*qTGW7.2b*^BG1.

Genes	Construction	Genome position	NIL-*qTGW7.2b* XLJ	NIL-*qTGW7.2b* BG1	Variation
LOC_Os07g39740	Promoter region (1999bp)	941	T	C	SNP
	Intron 3	3125	G	T	SNP
		3253	A	–	SNP
	Terminator region	4916	C	T	SNP
LOC_Os07g39750	Promoter region (1876bp)	11	–	C	SNP
		22	–	C	SNP
LOC_Os07g39780	5'-UTR	2102	C	A	SNP
	3'-UTR	8627	A	T	SNP
	Terminator region (912bp)	8844	A	G	SNP
		8852	A	G	SNP
LOC_Os07g39790	Promoter region (1830bp)	1609	G	T	SNP
	5'-UTR	1852	–	T	SNP
		1875	–	C	SNP
	CDS	2088	CGC (Arg)	CGG (Arg)	Synonymous
		2164-2466	AGC (Ser)	– – –	Deletion
		2233	TTG (Leu)	CTG (Leu)	Synonymous
LOC_Os07g39800	Promoter region (1831bp)	62	–	T	SNP
	Terminator region (1000bp)	5241	G	T	SNP
		5484	–	T	SNP
		5507	–	C	SNP
		5720	C	G	SNP

The horizontal line indicates base deletion; the red word represent mutations in the coding domain sequence.
